# Pharmacology under the microscope: the use of fluorescence correlation spectroscopy to determine the properties of ligand–receptor complexes

**DOI:** 10.1016/j.tips.2007.09.008

**Published:** 2007-12

**Authors:** Stephen J. Briddon, Stephen J. Hill

**Affiliations:** Institute of Cell Signalling, School of Biomedical Sciences, Medical School, University of Nottingham, Nottingham, NG7 2UH, UK

## Abstract

Recent years have revealed a high degree of structural organisation in the way in which cell-surface receptors and their associated signalling complexes interact at a molecular level. Fluorescence-based techniques have been at the forefront of methodologies used to investigate this organisation and dissect the pharmacology of drug–receptor interactions at the single-cell level. One such technique, fluorescence correlation spectroscopy (FCS), in conjunction with a fluorescent ligand or receptor, is capable of providing quantitative information about the number of receptors and their mobilities within small areas of the cell membrane that approach the size of some signalling domains. This article describes the use of FCS to perform subcellular quantitative pharmacology, with particular reference to G-protein-coupled receptors (GPCRs). In conjunction with other forms of fluctuation analysis, such as two-colour cross-correlation FCS and molecular brightness analysis, FCS provides the first opportunity to investigate the domain-specific nature of GPCR pharmacology.

## The need for single-cell pharmacology

The quantitative characterization of the interaction of a ligand with its receptor in terms of affinity and efficacy has been a mainstay of pharmacology since the concept of receptors was first introduced. Such parameters were derived initially from functional responses in isolated tissue samples and are now obtained routinely from populations of thousands of cells to give valuable information about ligand affinity, efficacy and binding capacity at a macroscopic level. The calculation of these ligand properties requires an accurate knowledge of both the free and bound concentrations of the drugs used. Mounting evidence suggests that membrane receptors and their associated signalling molecules exist in a highly ordered membrane environment [1–4]. This is particularly true of G-protein-coupled receptors (GPCRs), which can exist in dimeric and oligomeric complexes with other GPCRs, scaffolding proteins [such as A-kinase-anchoring proteins (AKAPs)] and adaptor molecules [such as β-arrestin and receptor-activity-modifying proteins (RAMPs)] ([1–5] and references therein). It is now evident that signalling from cell-surface receptors is highly compartmentalised, with receptors existing in close proximity to particular G-proteins, their effectors and the enzymes responsible for second-messenger metabolism [1,2]. Such organisation is mediated by protein–protein and protein–lipid interactions and is orchestrated by an association with the cytoskeleton and the tendency of some proteins to partition into membrane microdomains, such as lipid rafts and caveolae [2,3]. This leads to the formation of signalling microdomains, such as those described for receptors that stimulate the formation of cAMP. Here, local second-messenger concentrations are controlled by the proximity of adenylyl cyclase, phosphodiesterases and cAMP-activated protein kinase A [2,5]. A similar situation has been described for receptors linked to increases in intracellular calcium mediated by transient receptor potential (TRP) channels [4].

The movement of GPCRs to and from such domains can also be initiated by receptor stimulation. For instance, the A_1_-adenosine receptor moves out of caveolae following agonist stimulation, whereas, conversely, studies show that muscarinic, β-adrenergic and bradykinin receptors move into caveolae following activation ([1,3] and references therein). Similarly, the movement of receptors to clathrin pits can be initiated by agonist-mediated receptor phosphorylation and the binding of adaptor proteins, such as β-arrestin, adaptin and dynamin [6]. The formation of such macromolecular complexes not only leads to the movement of the receptor to clathrin-coated pits and its subsequent intracellular trafficking but can also mediate the activation of G-protein-independent signalling pathways, such as those leading to extracellular signal-related kinase (ERK) activation [1,5].

Alternative coupling to different effector signalling pathways can also have a major impact on the potency and efficacy of particular ligands [6]. For example, the β-adrenoceptor ligand propranolol is an inverse agonist of β_2_-adrenoceptor-mediated cAMP accumulation but activate ERK1/2 through the same receptor in the same cells [7,8]. It is, therefore, likely that the pharmacology of GPCRs can differ markedly depending on their membrane localisation and the signalling proteins present within that locality. If GPCRs can exist in different signaling domains within a single cell, then this will have important implications for the orchestration of drug action within a given cell type and the potential for cross-talk with other signalling pathways [9]. There is therefore an urgent need to develop techniques that can study the molecular pharmacology of GPCRs within specific membrane domains of single cells.

Fluorescence-based imaging technologies have been used widely to assess the cellular distribution of GPCRs and their associated signalling partners at a single-cell level [10]. In particular, genetic tagging of either the receptors themselves or associated proteins with green fluorescent protein (GFP) has proved a powerful technique for assessing the co-localisation of receptors and specific raft markers. Confocal-based techniques, such as fluorescence recovery after photobleaching (FRAP) [11], have provided useful information about the confinement of GPCRs within membrane domains, their macroscopic diffusion coefficients and how these are altered by receptor stimulation (e.g. [12]). More sophisticated techniques, such as fluorescence (Förster) or bioluminescence resonance-energy transfer (FRET/BRET), have been used to probe the proximity of receptor–receptor and receptor–effector interactions directly (reviewed in [10]). However, none of these techniques has both the temporal and spatial resolution to determine the properties of ligand–receptor complexes within membrane microdomains. In addition, they lack the ability to quantify the concentrations of both free and bound ligand, which are a prerequisite to the determination of potency and affinity.

Fluorescence correlation spectroscopy (FCS) is one technique with the potential to fill this gap [13–15]. As with the earlier described techniques, FCS is noninvasive and can be used on specific cell types within a mixed cell population, making it ideal for use with small numbers of cells (e.g. in samples obtained from biopsies). However, it also has a resolution high enough to localize measurements to areas of the cell membrane as small as some membrane microdomains. Uniquely, it is quantitative and its wide dynamic range allows the simultaneous detection of both fast- and slow-moving species (e.g. both free and receptor-bound ligand). Here, we discuss the application of FCS-based techniques to measuring the subcellular pharmacology of GPCRs.

## FCS and its application to studying ligand–receptor binding

The technique of FCS is based on measuring fluctuations in fluorescence intensity as fluorescently labelled particles diffuse through a small defined detection volume, which is approximately 0.25 fl in size (roughly the same volume as a mitochondrion) (Box 1). Statistical analysis of these fluctuations using autocorrelation analysis gives information about both the diffusion coefficient of the fluorescent particles and their concentration. The concept of correlation spectroscopy and the statistical principles involved were originally described in the 1970s. However, it was not until the advent of high-quality optics and photon-detection technology in the 1990s that the true power of the technique was finally realized (see [15] for a history of FCS development). This subsequently led to the application of FCS to the study of ligand–receptor interactions, by virtue of its ability to distinguish between species based on the speed of their diffusion (Box 2). Such a difference is present during ligand–receptor interactions because the diffusion co-efficient of a fast-moving free fluorescent ligand just above the cell surface decreases significantly when it binds to a much slower moving membrane receptor.

FCS has been used to characterise the binding of ligands to GPCRs, tyrosine-kinase receptors and ion channels [16–21]. Ligand-binding affinities, receptor densities and off rates for a number of different ligand–receptor combinations have all been determined successfully. In each case, even in heterogeneous cell populations from primary sources, useful quantitative information about ligand–receptor interactions was obtained, supporting the applicability of such methodology to studying pharmacology in biopsy samples [18,19,22–25]. In the case of the insulin C-peptide [18,22,23], kavain [25] and glucocorticoid receptors [26,27], the FCS data provided the first direct evidence for the existence of specific cell-surface GPCRs for these ligands.

### FCS using purified receptors

Ligand–receptor interactions were first quantified by FCS using a fluorescently labelled version of α-bungarotoxin to label the detergent-solubilised nicotinic acetylcholine receptor from *Torpedo californica* [28]. Although this study was performed on purified protein, it established the principle that FCS was capable of distinguishing free ligand from bound in a single measurement, based on the differences in their diffusion coefficients. A number of other similar studies have analysed binding to either membrane vesicles (e.g. to endothelin and transferrin receptors) or purified receptors [e.g. the 5-hydroxytryptamine-3 (5-HT_3_) receptor] [29–31]. This approach, coupled with molecular brightness analysis, is the basis for the use of fluorescent-fluctuation techniques in high throughput screening [32]. In this application, the slower diffusion of individual membrane vesicles, and the increase in molecular brightness as ligand molecules bind to each vesicle, forms the basis for identifying and separating the receptor-bound and free-ligand components. However, the real power of FCS is in its application to studying ligand–receptor interactions, in conjunction with imaging technology, in single cells.

### FCS measurements of receptor-fluorescent protein fusions in living cells

Since cell-based FCS meaurements have become feasible, one of the most widely used labelling strategies has been to genetically tag the protein of interest with GFP or a related fluorescent protein. Several studies have demonstrated that FCS can measure diffusion of a tagged receptor in the cell membrane directly and yield information about its membrane environment. With respect to GPCRs, the membrane diffusion of the complement C5a [33], bradykinin-BK_2_ [34] and A_1_-adenosine receptors [35] have all been characterised, as well as the tyrosine kinase-linked epidermal growth factor (EGF) receptor [36]. For the A_1_-receptor, a single diffusing species was identified, which was identical to that found for the diffusion of the antagonist-occupied receptor (see later) [35]. The heterogeneous diffusion of the other receptors was described variously in two to three different populations, suggesting the presence of the receptor in a number of different membrane environments [33,34].

### Agonist-receptor binding in single cells

In the majority of examples in which fluorescent ligands have been used to monitor receptor properties, agonist peptide ligands have been used to provide information on the diffusional characteristics of agonist-occupied receptors. Generally, this is because endogenous peptides (e.g. insulin and galanin) can be labelled readily. In addition, the extracellular-facing binding site of the receptor is sufficiently accessible to the ligand that there is less chance of the fluorophore interfering with its pharmacological action. However, in the case of GPCRs that have low molecular weight ligands, the design of fluorescent ligands is more complex because the binding site is often located within the transmembrane regions of the receptor [37]. In many cases, the addition of a fluorophore as large as the ligand itself creates a pharmacological entity that differs significantly in both potency and efficacy to the parent compound [35,37,38]. Choice of the correct fluorophore (both from a photophysical and pharmacological perspective) and an appropriate linker can be crucial to maintaining functional activity [24,30,38].

All FCS ligand-binding studies undertaken to date indicate that agonist-occupied receptors can exist in at least two forms within the cell membrane (indicated generally by τ_D2_ and τ_D3_, with τ_D1_ representing free ligand; Box 2, Table 1). These forms might represent receptor present in different membrane domains or associated with different cytoskeletal or scaffolding proteins. They might also be indicative of different functional forms of the ligand–receptor complex. The actual diffusion coefficients obtained for these species vary among studies, although their dwell times within the confocal volume fall generally within the 1–10 ms range for the faster species (τ_D2_) and more than 50 ms (τ_D3_) for the slower species (summarized in Table 1). It should be noted, however, that each of these two ‘bound’ components might actually be a composite of multiple species with differing diffusion coefficients. However, the relative amounts of these components differ significantly among receptor types. For instance, the agonist-occupied galanin receptor exists predominantly as τ_D2_ (88%) [19], whereas tetramethyl rhodamine (TMR)–EGF binding consisted largely of τ_D3_ (75%) [17]. Binding of two different fluorescent A_1_-receptor agonists (ABA- and ABEA-X-BY630) to the A_1_-receptor consisted of approximately equal amounts of each component [38,39]. Interestingly, a much higher percentage of the slower-diffusing τ_D3_ component was observed using the same ligand at the closely related A_3_-adenosine receptor expressed in the same cell type [40]. There is some circumstantial evidence to suggest that these diffusion components might relate to different functional forms of the receptor. For example, when FCS was used to measure the binding of labelled insulin [16], EGF [17] or kavain [25] to their respective receptors, different affinity states of the receptor appeared to be associated with the different diffusional components. Similarly, when a fluorescent dexamethasone derivative was used to detect cell-surface glucocorticoid receptors in a pituitary cell line, increasing the concentration of the ligand in a range approaching its measured affinity resulted in a shift from predominantly τ_D2_ to predominantly τ_D3_ [26,27]. In addition, the non-raft marker, DiO, showed a diffusion time comparable with that for τ_D2_, indicating that the cell-surface glucocorticoid receptor might move from non-raft domains to a more rigid raft-like domain on agonist activation. Some evidence also exists for the conversion of one diffusional component into another following more prolonged agonist stimulation (e.g. the β_2_-adrenoceptor), which might be related to receptor clustering prior to internalisation [20,41].

### Antagonist-receptor binding

**Figure 1:**
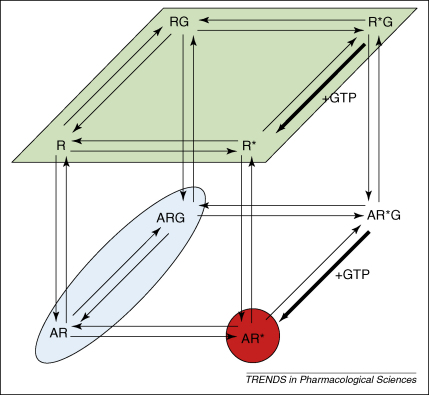
Using a range of labelling techniques enables subpopulations of receptor complexes to be studied. This diagram, based on the extended ternary-complex model, illustrates the receptor species likely to be present in a cell expressing a receptor (R) and a GTP-binding protein (G) in the presence of a ligand (A). The receptor can be present in either inactive (R) or active (R*) conformations, each of which can be found bound to G-protein (RG and R*G), ligand (AR and AR*) or both (ARG and AR*G). The coloured indicators show the species that are most likely to be detected when different labelling strategies are used for FCS experiments (see text). Green highlights directly tagged GPCRs (e.g. with GFP), which will represent the total ligand-unoccupied receptor population. Blue highlights the low concentrations of ligand used in FCS, at which an inverse agonist is likely to label predominantly the inactive forms of the receptor (R and RG). Red highlights that, similarly, at such low concentrations, an agonist ligand will detect mainly activated receptor (R* and R*G). However, because the intracellular concentration of GTP is high, any R*G formed will revert rapidly to R* and this will be the predominant form detected.

Two studies have used antagonist ligands to measure cell-surface receptor diffusion. For the 5-HT_3_ receptor, antagonist-occupied receptors were also detected in fast- and slow-moving forms [42]. The diffusion coefficients obtained appeared to correlate directly with receptor location and the slow-moving component (τ_D3_) was observed predominantly when the FCS detection volume was positioned over receptors that appeared clustered on the cell surface. The antagonist-occupied A_1_-receptor was described predominantly by a diffusion component (τ_D2_) of 15 ms. This was the same as that obtained for the tagged receptor itself [35]. Our studies using FCS to study the diffusion of the ligand-occupied A_1_-receptors are the only ones to have used both an agonist and antagonist ligand in the same cell line [35,38,39]. They appear to reveal significant differences between the diffusional properties of the agonist- and antagonist-occupied receptor populations, with the diffusion coefficients for both τ_D2_ and τ_D3_ of the agonist-occupied receptor being significantly faster than those obtained with the antagonist. In addition, in the case of the agonist, both of these components appeared to be equally sensitive to the displacement by competitor, whereas, for the antagonist, τ_D3_ appeared to be largely resistant to competitor. However, the most probable explanation for these differences is that agonist and antagonist ligands, under the conditions used in these FCS experiments, are labelling different functional forms of the receptor (see later and Figure 1).

## Progressing toward domain-specific pharmacology

For FCS to achieve the goal of assigning populations of occupied receptors with different diffusion coefficients to specific functional domains, signalling complexes or lipid rafts, the basic ligand-binding techniques described here need to be extended. Fortunately, advances in FCS technology, data analysis and labelling technologies in recent years suggest that this is certainly possible.

### Localising measurements to specific domains

Precise positioning of the FCS measurement volume over defined receptor clusters is possible using confocal imaging, as has already been demonstrated for ligand binding measurements on the 5-HT_3_ receptor [42]. It is, therefore, possible to use domain markers (separated spectrally from the ligand or receptor tag) to localise measurements to the domains of interest and to characterise diffusion of the receptors that are co-localised. Such markers have been used previously in conjunction with FCS in artificial systems (e.g. [43]). More recently, they have been used to label membrane domains in living cells and, subsequently, to measure domain diffusion [43–45]. For instance, diffusion of lipid-based raft and non-raft markers differed significantly in both RBL and COS-7 cells [43–45]. GFP-tagged transmembrance proteins or receptors, known to localise to specific membrane domains, have also been used as domain markers. For instance, a transferrin receptor–GFP fusion protein (non-raft) showed significantly faster diffusion than GFP–Thy1 (known to localise to rafts) [44]. Fluorescently labelled cholera toxin B-subunit (CTx-B) has also been used to label rafts in live cells [43,46]. Interestingly, the diffusion coefficients obtained in these experiments are of the same order of magnitude as those found for ligand-occupied receptors in the FCS studies. These studies also illustrate how the slow movements of strongly anchored domains are not detected easily using fluctuation techniques [43]. For example, when the membranes of RBL cells were labelled with fluorescent CTx-B, a strong fluorescence signal was detected, but diffusion of the label could only be seen when the cytoskeleton was disrupted [43,46].

This inability to detect effectively immobile particles, such as those anchored strongly to the cytoskeleton, is a limitation of standard FCS analysis. It stems from the essential requirement of autocorrelation analysis to have fluctuations in fluorescence over time, which are not present if there is limited diffusion within the detection volume. This can also limit the use of FCS in situations in which there is a large background population of fluorescent but immobile receptors. Detection of fluctuations on such a high background count can be difficult. Techniques such as image correlation spectroscopy (ICS) and raster ICS both use statistical analysis of images to obtain information about particle diffusion. The analysis used in ICS is more readily applicable in the presence of a high background and can, in some cases, actually provide an estimation of an immobile fraction [47,48]. Information on immobile fractions can also be provided on a macroscopic scale by techniques, such as FRAP. The recent development of scanning FCS (sFCS) might also help in the detection of very slow moving species [49–51]. Scanning FCS uses a moving illumination volume, scanned over the sample at a fixed velocity, enabling the detection of fluctuations from multiple points on its orbit. Autocorrelation analysis of sFCS data thus enables us to see if the signal is still present each time the beam returns to the same point. Because the orbital velocity of the beam is relatively slow (ms to s), this enables the detection of very slow moving species much more efficiently than standard single-point FCS. As an example, the application of sFCS to studies on the GFP-tagged bradykinin BK_2_ receptor, enabled the more accurate determination of a very slow moving receptor population that was not detected using single-point FCS measurements [34].

### Measuring specific receptor species in isolation

Comparisons of domain diffusion with those of ligand-occupied receptors or receptor–GFP fusion proteins provide some evidence for differences in the localization of, for instance, agonist- and antagonist-occupied receptors. However, simply showing that a receptor species and a domain have the same diffusion properties does not definitively place the species within that domain. The assignation of a species to a particular diffusion time is complex because differing domains might possess similar diffusion times and it is unlikely that a specific receptor species will be present in a given domain exclusively. Is it possible, therefore, to use FCS-based techniques to monitor specifically the diffusion of particular functional forms of the receptor or specific receptor–protein complexes?

As illustrated in Figure 1, the particular method used for labelling the receptor in the cell membrane already gives some degree of specificity in terms of the species of receptor that is detected. For instance, in terms of the cubic ternary complex model of GPCR activation [52], using a GFP-tagged version of the receptor itself will provide information about the properties of the total unoccupied receptor population (R, R*, RG and R*G; see Figure 1 for definitions). By definition, FCS uses low concentrations of fluorescent ligands and therefore operates at low receptor occupancies. In the case of an inverse agonist ligand, this will lead primarily to the detection of AR and ARG. Likewise, in intact cells in which the GTP concentration is high, low concentrations of agonist will detect AR* predominantly. This could explain some of the discrepancies seen in Table 1 but could also indicate differences in membrane properties of these receptor species. Allocation of such species to the τ_D2_ and τ_D3_ values obtained becomes more feasible in these situations. In particular, the use of a range of receptor ligands, both agonist and inverse agonist, with varying efficacies and potencies should enable the delineation of τ_D2_ and τ_D3_ and their assignment to different functional forms of the receptor.

Some newer and more advanced versions of fluorescence-fluctuation techniques might also help in identifying the diffusion properties of specific receptor–ligand and receptor–protein complexes. The first of these, two-colour cross-correlation FCS (FCCS), uses two overlapping detection volumes, usually provided by two co-focussed lasers of differing wavelengths [14,53]. Emission from two spectrally distinct fluorophores is separated into two detection channels and the fluorescence fluctuations in each can be autocorrelated. In addition to this, however, fluctuations can be correlated between the two channels (cross-correlated). A cross-correlation signal is therefore only obtained when both fluorescent species are present in the volume at the same time and gives an indication of the two species interacting. This enables both the diffusion coefficient and concentration of the dual-labelled species to be determined. Unlike FRET, for instance, FCCS does not rely on the proximity of the interacting components to obtain a signal but simply their co-diffusion. FCCS has been used in a number of single cell-based studies, including tracking the dissociation of cholera-toxin subunits [46], monitoring the interaction of calmodulin and Ca^2+^/Calmodulin-dependent protein kinase II (CaMKII) [54,55], detecting the interaction of the tyrosine kinase Lyn with the Fcɛ receptor [56], investigating immediate early gene dimerisation [57] and following caspase-3 activity [58,59]. When applied to the study of GPCRs, FCCS will be useful in the specific detection of ligand-occupied receptors and in detecting receptors interacting with signalling partners (e.g. G-proteins), scaffolding proteins (e.g. clathrin, caveolin) or other receptors (e.g. dimerisation). For instance, somatostatin analogues labelled with different fluorophores were used to obtain a cross-correlation signal at the cell membrane, which indicated the presence of receptor dimers [60]. FCCS is difficult to perform technically but recent improvements in FCCS methodology will improve the applicability of this technique to cell-based applications [61–63]. For instance, in single-wavelength excitation FCCS, a single excitation volume is used to excite both fluorescent species, ensuring 100% overlap in the excitation volumes. Single-wavelength FCCS has been used recently to investigate the proportion of dimer formation of the EGF receptor in living cells [64].

A further method that has been developed recently, bimolecular fluorescence complementation (BiFC), could also prove a valuable tool in isolating the diffusion properties of particular receptor complexes (reviewed in [65]). BiFC uses two non-fluorescent fragments of a fluorescent protein, such as yellow fluorescent protein (YFP), which regain their fluorescent properties when reconstituted. Fusion proteins made containing the non-fluorescent N- and C-fragments are constructed, such that fluorescence is only seen when the two proteins are in close enough proximity that reconstitution by complementation of full-length fluorescent protein is obtained. This enables the detection of specific fluorescent species with a high signal-to-noise ratio and has already been used to monitor the diffusion of GPCR oligomers [66] and to localise specific combinations of G-protein β and γ subunits [67,68].

FCS and FCCS are based on the temporal analysis of the fluorescence fluctuations, thus providing information about the dwell times of species within the detection volume. Diffusional analysis is limited to some degree, however, because at least a 1.6-fold change in diffusion time (corresponding to a 6-fold change in mass) is required to reliably differentiate between two species [69]. This would not distinguish, for instance, between monomeric and dimeric species of GPCRs based on mass difference alone. Analysis of the fluctuations with respect to the brightness of the fluorescent species, by either photon-counting histogram analysis (PCH) [70] or fluorescent-intensity distribution analysis (FIDA) [71], however, affords information about particle number and molecular brightness. This can distinguish heterogenous species that differ by only twofold in their molecular brightness (e.g. monomer vs dimer) [72]. The existence of a range of oligomer sizes for the EGF receptor and the effect of cholesterol on this distribution has been demonstrated recently using FIDA [73]. In other examples, PCH analysis has been used to show the formation of higher-order oligomers of the Na^+^/H^+^ co-transporter [74], whereas similar analysis detected dimeric species of membrane-bound adenylate kinase [75]. In a similar manner, PCH has been used to monitor aggregation of the retinoid receptor and to determine the stoichiometry of its binding [76,77]. More recently, the use of two-colour PCH in cells raises the possibility that multiple species could be monitored simultaneously using this technique [78].

## Conclusions

FCS has already proved a powerful technique for quantifying the diffusion of receptors and ligand–receptor complexes in small areas of living cell membranes. It gives us new information about the distribution and organisation of membrane receptors and enables such analysis to be performed in heterogeneous cell populations. A number of recent advances in FCS technologies, such as total internal reflection-FCS [79–81] and the use of nanostructures [82], could further increase the axial resolution of these measurements. Coupled with the newer labelling technologies described and the availability of fluorescent ligands with a wider range of affinities, the delineation of the properties of specific receptor–ligand and receptor–protein complexes at a subcellular level is a real possibility.

## Figures and Tables

**Figure I:**
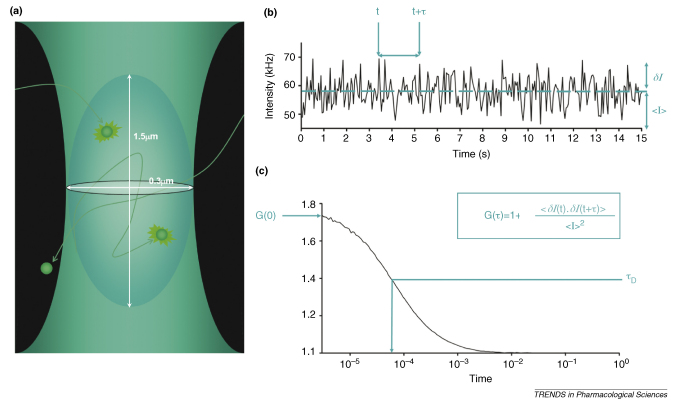
**Basic principles of FCS. (a)** Schemative representation of the confocal detection volume. **(b)** Variations in the detected fluorescence intensity with time. **(c)** Autocorrelation analysis of fluorescence fluctuations (see text for details).

**Figure I:**
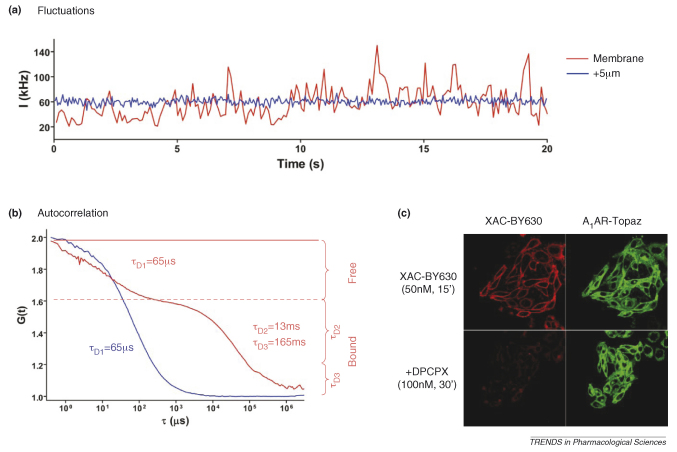
**(a)** Fluorescence fluctuations obtained above (blue trace) and on the membrane (red trace) of CHO cells expressing the human A_1_-adenosine receptor following incubation with a fluorescent receptor antagonist, XAC-BY630. **(b)** Corresponding autocorrelation analysis of the fluorescence fluctuations obtained. **(c)** Confocal images of CHO cells expressing a fluorescently (Topaz)-tagged variant of the A_1_-adenosine receptor, incubated with XAC-BY630 in the presence and absence of the non-fluorescent receptor ligand DPCPX, showing a high degree of specific binding of the fluorescent receptor antagonist.

**Table 1:** Summary of receptor–ligand-binding studies using FCS

Receptor class	Receptor	Ligand	Activity	Cells	Component 1 (τ_D2_; ms, %)	Component 2 (τ_D3_; ms, %)	Refs
**7TM**	Adenosine-A_1_	XAC-BY630	Antagonist	CHO (hamster ovary)	17 (85%)	321(15%)	[35]
	ABA-BY630	Agonist	CHO	8 (48%)	233 (52%)	[39]
	ABEA-BY630	Agonist	CHO	9 (40%)	267 (60%)	[38]
	A_1_-AR-Topaz	Receptor	CHO	15 (100%)		[35]
Adenosine-A_3_	ABEA-BY630	Agonist	CHO	6 (25%)	135 (75%)	[40]
Bradykinin-BK_2_	BK_2_R-YFP	Receptor	HEK (human kidney)	16 (n.d.)^a,b^	140 (n.d.)^a,b^	[34]
Complement-C5a	C5aR-YFP		HEK	2 (100%)^c^		[33]
β_2_-adrenoceptor	AF532-arterenol	Agonist	Rat neurones	1.8 (78%)	160 (22%)	[20]
			A549 cells (alveolar)	3 (66%)	45 (33%)	
			C6 cells (glioma)	0.7 (65%)	9.4 (35%)	
Galanin	Rh–galanin	Agonist	Rinm5F (rat insulinoma)	22 (88%)	700 (12%)	[19]
**Tyrosine kinase**	Insulin	Rh–insulin	Agonist	Human renal tubular	0.8 (51%)	20 (49%)	[16]
EGF	Rh–EGF	Agonist	Human fibroblasts	3 (24%)	100 (76%)	[17]
	EGFR-mRFP	Receptor	CHO	54 (100%)		[36]
**Ion channel**	GABA_A_	AF532-muscimol	Allosteric modulator	Rat neurons	4 (79%)	72 (21%)	[22]
	AF532-Ro07-1986/602	Allosteric modulator		7 (n.d.)	360 (n.d.)	[25]
5-HT_3_	Cy5-GR119556	Antagonist	HEK293	1–10 (n.d.)	>20 (n.d.)	[43]
**Misc.**	Glucocorticoid receptor	FITC–dexamethasone	Agonist	AtT20 (mouse pituitary)	4 (25%)	250 (65%)	[27,28]
C-peptide	Rh–C-peptide	Unknown	Human renal tubular	1 (17%)	80 (83%)	[18]
IgE	AF488-IgE	Activator	RBL (mast cells)	80 (100%)^d^		[56]

The table summarises the ligand–receptor species detected in FCS experiments using a variety of receptors and ligand types (n.d. = not determined). ^a^Other experiments in this study using scanning FCS indicate τ_D2_ = 72%, τ_D3_ = 12%, with 16% consisting of a slower diffusing third component. ^b^Diffusion times are estimates calculated from published diffusion coefficients (D), using the equation τ_D_ = r^2^/4D, where r = 0.15 μm, estimated from the excitation wavelength (488 nm). ^c^As for b, except r = 0.17 μm (514 nm). ^d^As for b, except the equation used was τ_D_ = r^2^/8D, as multiphoton excitation was used, with r = 0.25 μm. 7TM, seven transmembrane spanning receptor; BK, bradykinin; FITC, fluorescein isothiocyanate; GABA, γ-amino butyric acid.

## Box 1 Basic principles of FCS and autocorrelation analysis

FCS uses a small detection volume. This is created by focussing a laser to a diffraction-limited spot using a microscope objective lens with a high numerical aperture. Positioning of a pinhole in the confocal image plane creates a Gaussian-shaped detection volume of approximately 0.25–0.5 fl, depending on the excitation wavelength [e.g. half axes of 0.15 (lateral) and 0.75 μm (axial) for 488 nm excitation through a 1.2 NA objective lens, with the pinhole adjusted to 1 Airy unit diameter]. As fluorescent molecules diffuse through this volume, they are excited and the emitted photons are detected in a time-correlated manner using a single photon-counting device, such as an avalanche photodiode. Over time, this leads to fluctuations in the detected fluorescent intensity. These fluctuations can be caused by translational diffusion of the fluorescent species through the volume but also by photophysical events within the fluorophore or changes in its fluorescent properties, such as quantum yield or interstate conversions (e.g. caused by conformational changes in the labelled species) [13–15]. Autocorrelation analysis is a way of analyzing the time-dependent changes in the amplitude of these fluctuations about the mean fluorescent intensity. Autocorrelation analysis compares the size of the fluctuation (δI) from the average intensity (<I>) at time t with a subsequent fluctuation at a time t + τ later. This determines effectively whether the species responsible for the fluorescence is still present in the volume. Using a range of values for τ, results in an autocorrelation function, G(τ), which is usually normalized to the square of the mean intensity (<I>). This function yields information about the average dwell time (τ_D_) and the average number of molecules (N) of the fluorescent species within the volume during the measurement. The dwell time (τ_D_) is obtained from the mid-point of the decay of the autocorrelation function, G(τ). Knowledge of the size of the detection volume (acquired using a standard of known diffusion coefficient) can therefore yield the diffusion coefficient of the species, which is related to properties, such as molecular mass, hydrodynamic radius and viscosity of the sample. In addition, because τ→0, G(0)→1/N, where N is the average number of particles within the detection volume during the measurement. From this value, using knowledge of the shape and dimensions of the detection volume, the absolute concentration of the detected species can be calculated readily. ‘N’ and ‘τ_D_’ are obtained from the autocorrelation function by non-linear curve-fitting of the data to an appropriate biophysical model, with the choice of model (e.g. 2D vs 3D, free vs anomalous diffusion) being crucial to the correct quantification and interpretation of the data. With modern hardware and detection technology, FCS can provide useful information over a large range of fluctuation times (1 μs–500 ms) and has thus led to its application in a number of fields, ranging from photo-physical investigations and chemical reaction monitoring to cell-based studies on protein diffusion. The inverse relationship between the autocorrelation function at time zero [G(0)] and the number of diffusing particles (N) results in a greater sensitivity of the technique at lower particle numbers, such that the concentration range that can be used practically with FCS is between 0.1 and 400.0 nM, depending on signal-to-noise ratio of the sample [corresponding to an average particle number (N) of 0.01–40.0]. This signal-to-noise ratio, particularly in cells, can be severely affected by non-correlated background signal, such as shot noise or autofluorescence, although this can be accounted for in the biophysical model used. Further artefacts can be introduced by not allowing for the photophysics and photobleaching of the fluorescent label (Figure I).

## Box 2 FCS enables the distinction between free and bound ligand in small areas of cell membrane

In complex mixtures, in which two fluorescent species with differing diffusion coefficients are present, their autocorrelation functions (Box 1) are simply additive, resulting in a biphasic decay curve for G(τ). This can yield diffusion coefficients for both species and also enables the calculation of their individual concentrations from their relative contributions to the amplitude of the function. It is this property of the analysis that proves useful for investigating ligand–receptor interactions. A free ligand molecule in the vicinity of the cell membrane will show fast 3D Brownian diffusion, at a rate corresponding to its molecular mass. If that ligand binds subsequently to a slow-moving membrane-localised receptor, its diffusion coefficient will be reduced substantially. Therefore, placing the FCS detection volume on the cell membrane will yield an autocorrelation function composed of a fast-moving component (representing the free ligand diffusion) and one or more slow-moving components (representing the membrane-bound ligand) [83]. This is illustrated (Figure I) with fluctuation data obtained on the upper membrane of CHO cells expressing the human A_1_-adenosine receptor labelled with a fluorescent antagonist that binds to this receptor (XAC-BY630) [35]. Fluorescence fluctuations obtained 5 μm above the cell are produced by fast-moving free ligand alone. Autocorrelation analysis of these fluctuations results in a monophasic decay curve, with a diffusion time showing a single fast-moving component. The average dwell time (τ_D_) of a particular species is determined from the mid-point of the decay of the autocorrelation function [G(τ)]. For example, for the diffusional characteristics of the free ligand in the figure, τ_D1_ is 65 μs. Fluctuations recorded when the volume is placed in a position incorporating both extracellular solution and cell membrane are quantitatively and qualitatively different in their amplitude and temporal nature. This is owing to the presence of slower moving receptor–ligand complexes (and possibly non-specifically bound ligand). Autocorrelation analysis now reveals a more complex curve showing additional slow moving components representing bound ligand (τ_D2_ and τ_D3_). Fitting of the autocorrelation function to an appropriate biophysical diffusion model through non-linear curve-fitting enables the proportion of each component present to be calculated. This information can then be used to calculate the actual concentrations of free ligand and ligand-occupied receptor within the measurement volume. The quantitative biophysical model used for the curve fitting needs to be appropriate for the system. In this case, the model used assumed free 3D diffusion of the free ligand and free 2D diffusion of the bound components. The fitted equation also incorporated terms for particle number, photo-physical fluctuations from the fluorophore and the geometric shape of the volume. Such quantification also relies on a number of other conditions, for instance, low background noise and autofluorescence. In particular, the free and bound ligand should have identical molecular brightness (quantum yield) or the determination of N is skewed heavily toward the brighter species. Under these conditions, FCS measurements are therefore capable of yielding the true free concentration of ligand in the immediate vicinity of the receptor, in addition to the diffusion coefficients and concentrations of any ligand-occupied receptors.
